# Epidemiologic aspects of the malaria transmission cycle in an area of very low incidence in Brazil

**DOI:** 10.1186/1475-2875-6-33

**Published:** 2007-03-19

**Authors:** Crispim Cerutti, Marcos Boulos, Arnídio F Coutinho, Maria do Carmo LD Hatab, Aloísio Falqueto, Helder R Rezende, Ana Maria RC Duarte, William Collins, Rosely S Malafronte

**Affiliations:** 1 Department of Social Medicine, Biomedical Center, Federal University of Espírito Santo, Av. Marechal Campos, 1 468, Maruípe, Vitória-ES, Zip Code: 29040-091, Brazil; 2 Department of Infectious and Parasitic Diseases, Faculty of Medicine, University of São Paulo, Av. Dr. Arnaldo, 455, Cerqueira César, São Paulo-SP, Brazil; 3 State Department of Health, Av. Marechal Mascarenhas de Moraes, 2 025, Bento Ferreira, Vitória-ES, Brazil; 4 Faculty of Public Health, Av. Dr Arnaldo, 715 Cerqueira César, São Paulo-SP, Brazil; 5 Division of Parasitic Diseases, Centers for Disease Control and Prevention, Atlanta,, Georgia USA; 6 Protozoology Laboratory, São Paulo Institute of Tropical Medicine, Av. Dr. Enéas de Carvalho Aguiar, 470, Cerqueira César, São Paulo-SP, Brazil

## Abstract

**Background:**

Extra-Amazonian autochthonous *Plasmodium vivax *infections have been reported in mountainous regions surrounded by the Atlantic Forest in Espírito Santo state, Brazil.

**Methods:**

Sixty-five patients and 1,777 residents were surveyed between April 2001 and March 2004. Laboratory methods included thin and thick smears, multiplex-PCR, immunofluorescent assay (IFA) against *P. vivax *and *Plasmodium malariae *crude blood-stage antigens and enzyme-linked immunosorbent assay (ELISA) for antibodies against the *P. vivax*-complex (*P. vivax *and variants) and *P. malariae*/*Plasmodium brasilianum *circumsporozoite-protein (CSP) antigens.

**Results:**

Average patient age was 35.1 years. Most (78.5%) were males; 64.6% lived in rural areas; 35.4% were farmers; and 12.3% students. There was no relevant history of travel. Ninety-five per cent of the patients were experiencing their first episode of malaria. Laboratory data from 51 patients were consistent with *P. vivax *infection, which was determined by thin smear. Of these samples, 48 were assayed by multiplex-PCR. Forty-five were positive for *P. vivax*, confirming the parasitological results, while *P. malariae *was detected in one sample and two gave negative results. Fifty percent of the 50 patients tested had IgG antibodies against the *P. vivax*-complex or *P. malariae *CSP as determined by ELISA. The percentages of residents with IgM and IgG antibodies detected by IFA for *P. malariae*, *P. vivax *and *Plasmodium falciparum *who did not complain of malaria symptoms at the time blood was collected were 30.1% and 56.5%, 6.2% and 37.7%, and 13.5% and 13%, respectively. The same sera that reacted to *P. vivax *also reacted to *P. malariae*. The following numbers of samples were positive in multiplex-PCR: 23 for *P. vivax*; 15 for *P. malariae*; 9 for *P. falciparum *and only one for *P. falciparum *and *P. malariae*. All thin and thick smears were negative. ELISA against CSP antigens was positive in 25.4%, 6.3%, 10.7% and 15.1% of the samples tested for "classical" *P. vivax *(VK210), VK247, *P. vivax*-like and *P. malariae*, respectively. Anopheline captures in the transmission area revealed only zoophilic and exophilic species.

**Conclusion:**

The low incidence of malaria cases, the finding of asymptomatic inhabitants and the geographic separation of patients allied to serological and molecular results raise the possibility of the existence of a simian reservoir in these areas.

## Background

There are 300 to 500 million new cases of malaria worldwide each year, and these result in 0,7 to 2,7 million deaths [1]. Almost all malaria cases in Brazil are reported from the Amazon region, with 459,013 cases in 2004 [2]. Outside the Amazon region, malaria is restricted to residual foci in areas where remnants of tropical forest can still be found. One such area is the state of Espírito Santo.

Espírito Santo is a costal state located in the Southeast of Brazil. Indigenous malaria is observed in the highlands, which are not more than fifty kilometres on average from the sea. Ten to thirty autochthonous cases are reported every year in an area of about 5,343 km^2^. The species diagnosis, based on thick smear examination, is reported as *vivax *malaria with low parasite count as a rule (less than 300 parasites/mm^3 ^of blood). There are no case clusters except for a few occasions on which two cases were registered in the same place at the same time. As transmission can hardly be explained by man-to-man dispersion in such a situation, it was decided to proceed to an investigation to clarify the possibility of there being an unrecognized reservoir and to determine more precisely the species involved. Additionally, plasma samples from infected patients and residents were screened with the objective of identifying antibodies against *Plasmodium vivax*-complex (*P. vivax *and variants) and *Plasmodium malariae*/*Plasmodium brasilianum *circumsporozoite protein (CSP).

## Methods

### Study area and population

**Figure 1:**
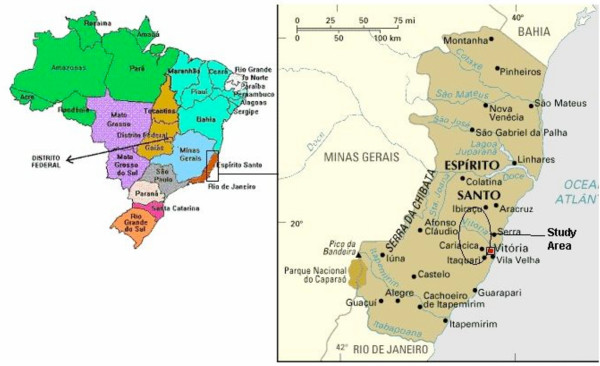
Location of the state of Espírito Santo in Brazil and the study area.

Indigenous malaria cases in the state of Espírito Santo are registered mainly in nine municipalities distributed in an oval-shaped area of about 5, 343 km^2 ^with a population of 215,000 inhabitants. These municipalities are located between 19.6° and 20.6° South latitude and 40.6° and 41° West longitude (Figure 1). The topography is irregular, with narrow valleys and small mountains with mean heights of around 800 meters. The climate is tropical, but lower temperatures of around 15°C occur during the winter months (from May to August) because of the high altitude. The economy is based on agriculture, with coffee, bananas and vegetables being grown on small farms. The tropical forest on the tops of hills is still quite preserved and is very close to human dwellings. The fauna consists of birds, reptiles and small mammals, including simians from the Cebidae and Atelidae families. Although anophelines of the Nyssorhynchus subgenus were found in these areas, *Anopheles darlingi*, which is incriminated in malaria transmission in the Amazon basin, is absent in this region. However, *Anopheles (Kerteszia) cruzii*, an acrodendrophilic species responsible for malaria transmission in the Atlantic Forest, which is outside the Amazon region, can be found in the forest but not near the houses. The population consists mainly of descendants of nineteenth-century German and Italian immigrants.

### Study design

Once a new case had been detected and after written informed consent had been signed, a questionnaire was filled out with demographic, occupational and environmental data. Five millilitres of venous blood were drawn in a vacuum tube with EDTA for thick and thin smear preparations, serology and PCR procedures. Blood was centrifuged at 300 g to separate the red blood cells from plasma and kept at -20°C until it was transported in dry ice by air to the Protozoology Laboratory at the São Paulo Institute of Tropical Medicine for processing. After enrolment, the patient was immediately treated according to Brazilian Ministry of Health standards for malaria therapy. For the 14^th ^to the 50^th ^patient enrolled in the study, each subject had his/her home visited by the research team, which also visited the subject's neighbours in an area of about two kilometres around the patient's home. Processing of neighbours' blood samples was the same as that described for the patients.

For serological control purposes, after written informed consent forms were signed, an additional 192 blood samples were collected in a malaria-free area located 50 km south of the southernmost patient's dwelling.

### Thick and thin-smear preparation and reading

Thick and thin smears were prepared according to the technique recommended by the World Health Organization [3]. The slides were read using a light microscope at 1,000 × magnification.

### CSP ELISA

Anti-CSP antibodies were detected using synthetic peptides corresponding to the *P. vivax*-complex (*P. vivax *and variants) and *P. malariae *circumsporozoite protein (CSP) reported in the literature [4-7].

The technique was performed according to Zavala et al. [8] and Curado et al. [9]. Cut-off values were taken as three standard deviations above the means of the absorbances obtained by testing 40 serum samples from blood donors who had never lived in malaria-transmission areas. The following values of cut-off were used: 0.178 for "classic" *P. vivax*, 0.297 for *P. vivax *VK247, 0.292 for *P. vivax*-like and 0.153 for *P. malariae*.

### Immunofluorescent Assay (IFA)

Ferreira and Sanchez's protocol [10] was followed with minor modifications. Crude blood-stage antigens were obtained from *P. vivax*-infected patients, *P. malariae*-infected monkeys and *P. falciparum *cultures. Cut-off values were 1:80 for the detection of both IgM and IgG antibodies and corresponded to the greatest dilution showing blood cell fluorescence in a panel of 40 serum samples from blood donors who had never lived in malaria-transmission areas. Because of the difficulty in obtaining parasitized blood from malariae malaria patients (*P. malariae *is sometimes difficult to distinguish from *P. vivax *on a thick smear), a number of slides of *P. malariae *parasites were kindly supplied by CDC/Atlanta. Only those patients who gave positive results in ELISA against *P. malariae *CSP were tested for antibodies against this species by IFA. Results were expressed as titres corresponding to the reciprocal of the last plasma dilution that gave a positive reaction.

### Multiplex PCR

After thawing, red-blood-cell pellets were submitted to DNA extraction according to instructions provided with the Wizard^® ^Genomic DNA (Promega) commercial kit with the following minor modifications: following addition of 1 μl 5% saponin, the blood was incubated at 37°C for five minutes and after incubation the pellet was washed with PBS until the supernatant became clear. All the other steps were carried out according to the instructions supplied with the kit. For amplification, the Rubio et al. [11] protocol was followed. The reaction was carried out in two steps to improve sensitivity. Species-specific primers were used to detect the three species responsible for malaria infection in Brazil, namely, *P. vivax*, *P. malariae *and *P. falciparum*. Plasmodial DNAs extracted from the *P. vivax *malaria patients attending the Superintendence for the Control of Endemics (SUCEN) in São Paulo or from *P. falciparum *cultures, were used as positive controls.

### Anopheline captures

The entomology team visited each of the transmission locations no more than seven days after the blood sampling procedure. The mosquitoes were captured at random between 6:00 p.m. and 10:00 p.m., both indoors and outdoors. The Castro apparatus was used to collect mosquitoes, and the specimens were identified in the Tropical Medicine Unit, Federal University of Espírito Santo, based on the method of Faran and Linthicum [12]. After identification, the specimens were stored in NUNC tubes filled with isopropyl alcohol, with each one containing only mosquitoes of the same species and location. The tubes were sent to the Protozoology Laboratory at the São Paulo Institute of Tropical Medicine to be tested for the presence of *Plasmodium *by PCR.

### PCR procedure used with the mosquito samples

DNA from each subset of a maximum of ten specimens from the same species and location was extracted according to the Oskam protocol [14], with the following minor modifications: after incubation at 60°C for three hours in lyses buffer, the samples were centrifuged at 10,000 g for 10 minutes. The supernatant was harvested and kept overnight at -20°C after addition of 0.1 volume of sodium acetate and two volumes of cooled 100% ethanol. The material was then centrifuged again at 10,000 g at 4°C for 10 minutes. The sediment was washed with 70% ethanol and resuspended in 50 μl of T.E. after drying. For amplification, the Kimura protocol [13] modified by Win *et al. *[15] was followed. Species-specific primers were used to detect *P. vivax*, *P. malariae *and *P. falciparum*.

### Data analysis

Continuous variables were expressed as means or medians in terms of their adequacy to normality. Categorical variables were expressed as percentages. Serological results were expressed as geometric mean titres or absorbance depending on the assay. Spearman's correlation test (two-tailed) was applied to verify the presence of correlation between ELISA absorbances or IFA titres and age. The same test was applied to determine whether there was a correlation between ELISA absorbances and both IgM and IgG IFA titres. The Kruskal-Wallis nonparametric test was applied to determine whether there was an association between ELISA absorbances or IFA titres (IgM and IgG) and the demographic patient variables occupation, occupational activities related to the rural environment and level of contact with the rural environment. Differences between proportions were assessed with the Chi-square test or Fisher Exact test, as indicated. A 5% significance level was used.

### Ethical clearance

The study was approved by the Ethical Committees of the Biomedical Centre, Federal University of Espírito Santo and the São Paulo Institute of Tropical Medicine.

## Results

### Epidemiologic data

From April 2001 to March 2004, sixty-five patients were enrolled in the study. Twelve were enrolled in 2001, 18 in 2002, 24 in 2003 and 11 in 2004 (until the end of March) (Table 1). Cases occurred throughout the year, with a small predominance in March and April. The percentages of cases in 2001 and 2004 were low because surveillance was not year-round in those years. Six cases occurred in 2001 before April, and 20 in 2004 after March (the last month of surveillance).

**Table 1:** Frequency of positive cases diagnosed as *P. vivax *malaria from April 2001 to March 2004.

**YEAR**	**Frequency of cases**	**Percentage (%)**
2001	12	18.5
2002	18	27.7
2003	24	36.9
2004	11	16.9

**Total**	**65**	**100.0**

The mean age of patients was 35.1 years. Most of the patients were males (78.5%) and 2/3 of them lived in rural areas (64.6%). Those who lived near towns or in the capital declared at least one visit to a rural area in the preceding 30 days. The most common occupations declared were farmer (35.1%) and student (14%), but most of the students help their families on the land both during seeding and harvest, thus increasing the figure for occupational contact with rural areas to 59.6%. Irrespective of occupation, some kind of frequent contact with the land was declared by 67.3% of the patients. Many of them lived and worked in towns but had a property in a rural area that they visited almost every day.

Almost 1/3 of the patients (30.8%) had not travelled in the preceding two years. Those who had travelled claimed not to have travelled to the Amazon region. One third of the patients (33.8%) had been living in the transmission area since they were born. Almost all the patients (95%) were experiencing their first episode of malaria. Only 36.9% of the patients declared having visited the forest, but this low percentage may represent an information bias as the local population is very afraid of environmental laws. The forest, however, is close to human dwellings, so that exposure is possible regardless of whether or not there are excursions into it. Table 2 shows the relationship between travel history and history of visits to the forest.

**Table 2:** Relationship between travel history in the previous two years and history of visits to the forest in patients with malaria in Espírito Santo state.

**Travel History**	**Visits to the forest**		**Total**
		
	**No**	**Yes**	
**None**	11	9	20
**To other municipalities in the transmission area**	11	3	14
**To non-endemic areas**	19	12	31

**Total**	**41**	**24**	**65**

Some kind of contact between the patients enrolled or between the patients and other malaria cases prior to the illness was found in 11 instances. Patients 2 and 3 were married to each other and developed symptoms at almost the same time. Patients 28 and 29 were related and declared a malaria case that had occurred five months before, 1 km from their house. Patient 30 was informed of a malaria case the previous month 15 km from his house. Patient 32 had contact with two malaria cases near his house two years previously. Patient 35 declared frequent contact with cases 28 and 29 and became ill three months after them. Patients 40 and 41, who were from the same geographic region, became ill at the same time. Patients 42, 44 and 45 became ill between June 9 and July 18, 2003. They lived close to one another. Case 51 also lived close to them but only became ill in November of the same year. Case 53 reported contact with a malaria patient who had arrived from the Amazon region 30 days before. Cases 52 and 59 were related but lived about 100 km apart and became ill within an interval of two months. Patients 55 and 56 were married to each other and became ill at the same time.

From March 2002 to November 2003, 1,777 residents from the affected locations were submitted to blood sampling, with a median of 46 samples collected for each case. The mean age of the residents was 31.3 years. Distribution by gender was equivalent (52.3% males; 47.7% females). Seventy-five percent of the residents (1,333 subjects) were born in the same municipalities where the cases had occurred. Of the remainder, 290 (16.3%) were born in other municipalities in the state and 144 (8.1%) in other states or countries. Information was not obtained for ten residents (0.6%). Only two subjects were born in the Amazon region. The median time of residence in the affected area was 16 years. Travel in the preceding two years was declared by 1,143 residents (64.3%), but only twelve of them (0.7%) declared that they had travelled to the Amazon region.

### Clinical data

The patients' symptoms were abundant and long-lasting but not severe. The interval between the first symptom and diagnosis was 13.9 ± 7 days. Fever was present in all the patients. It was periodic in 96.9% of the cases, occurring every 48 hours in 73.8% of the cases and every 24 hours in the remainder. Spleen was palpable in only 57.4% of the patients, measuring 2.7 ± 0.7 cm below the left costal margin.

The patients complained of several symptoms. Other signs and symptoms such as headache (87.7%), chills (86.2%), sweating (75.4%) and weakness (72.3%) were reported.

### Thick and thin-smear readings

Malaria parasites were found in the slides prepared with blood collected from the patients but not in those prepared with blood from the other individuals tested. Parasite morphology was compatible with *P. vivax*, with the enlarged red blood cells, typical stippling and enlarged amoeboid cytoplasm of the parasites.

Parasite count was low in the majority of patients (83.1%). A count between 300 and 500 parasites/mm^3 ^was observed in 7.7% of the patients, and the same percentage had a count between 500 and 10,000 parasites/mm^3^. One patient had a parasite count of 15,000 parasites/mm^3^. He had been submitted to splenectomy for treatment of hepatosplenic schistosomiasis several years previously.

### CSP ELISA

Of 50 patients tested for antibodies against CSP peptides, 25 (50%) reacted to at least one synthetic peptide. Several patients were positive for more than one peptide; 44%, 22%, 20% and 22% were positive for "classical" *P. vivax *(VK210), *P. vivax *VK247, *P. vivax*-like and *P. malariae*, respectively.

Of 1,702 samples (95.8% of the total) collected from the residents and tested, 615 (36.1%) reacted to at least one synthetic peptide. Several asymptomatic residents were positive for more than one peptide; 25.4%, 6.3%, 10.7% and 15.1% were positive for "classical" *P. vivax *(VK210), *P. vivax *VK247, *P. vivax*-like and *P. malariae*, respectively. Forty percent of the samples reacted for both variant VK 247 and *P. malariae *CSP peptides. Fifty-one samples (3%) were positive exclusively for *P. vivax*-like.

### IFA

Eleven patients were positive for *P. malariae *anti-CSP antibodies, but as this antigen was not available in sufficient quantity, only seven of them were assayed for detection of anti-blood stage antibodies by IFA. Seven patients were positive for IgM and IgG with a geometric mean titre (GMT) of 3,120 and 390 respectively. GMT for both IgM and IgG antibodies against *P. vivax *in the 50 patients were 1,457 and 5,761, respectively.

**Figure 2:**
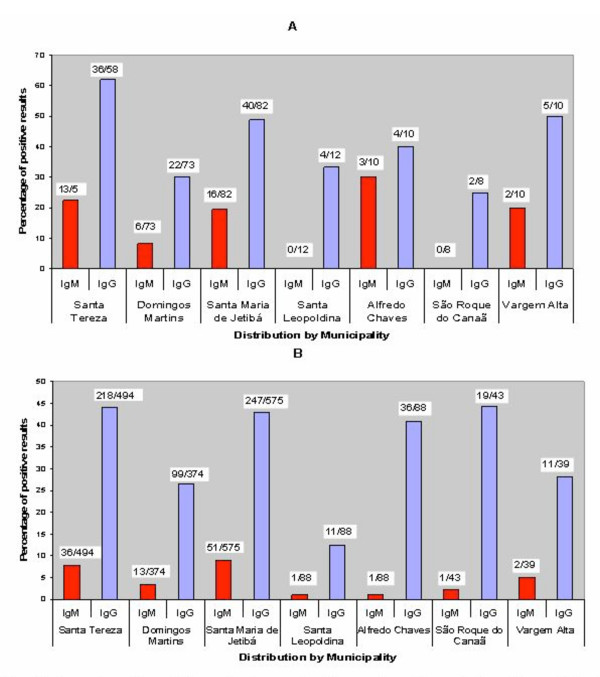
Distribution of positive IFA results for antibodies against *P. malariae *(A) and *P. vivax *(B) crude-blood-form antigens by municipality in the endemic area of the state of Espírito Santo.

Two hundred and fifty-three samples from residents were assayed for detection of anti-blood stage antibodies against *P. malariae *by IFA. Of these samples, 239 were chosen because they had reacted against CSP synthetic peptides in ELISA, and 14 had tested negative against *P. malariae *CSP. Of this subset, 72 samples (30.1%) were positive for IgM antibodies (GMT = 191.1) and 135 (56.5%) positive for IgG antibodies (GMT = 504.7). Antibodies against *P. vivax *crude blood stage antigens were assayed by IFA in 1,701 samples from the residents (95.8% of the total). Of these, 105 (6.2%) were positive for IgM antibodies (GMT = 206) and 641 (37.7%) positive for IgG antibodies (GMT = 313.3). As ten samples were positive for *P. falciparum *DNA by multiplex-PCR (see below), those samples and a randomized subset of the negative ones (182, or 10.2% of the total) were tested for antibodies against *P. falciparum *blood stage antigens by IFA. IgM antibodies were detected in four of the samples that were positive by multiplex-PCR and in 26 out of the 182 chosen at random (13.5%). The figures for IgG antibodies were three and 25 samples (13%), respectively. GMT was 101.8 and 116.8 for IgM and IgG antibodies, respectively. The percentages of positive results by IFA for *P. vivax *and *P. malariae *in the various municipalities in the transmission area are shown in Figure 2.

### Relationship between IFA titres, ELISA absorbances and demographic variables

A significant negative correlation was found among the patients between anti-*P. vivax *IFA IgM titres and age (p = 0.042) using Spearman's test. All the other correlations were not statistically significant at the 5% level. No significant association was found according to the Kruskal-Wallis nonparametric test between anti-*P. vivax *IFA titres (both IgM and IgG) or ELISA absorbances and the variables occupation, occupational activities related to the rural environment or level of contact with the rural environment.

A higher frequency of samples that were IgG-positive by IFA was found among male residents. The frequency found for *P. malariae *was 52% for males and 37.5% for females (p = 0.03); the corresponding figures for *P. vivax *were 40% and 35% (p = 0.03). A significant positive correlation was found between the time for which the subject had lived in the endemic area and IgG antibodies against *P. vivax *by IFA using Spearman's test (p = 0.05). None of the other correlations reached the significance level. While there were more positive results against "classical" *P. vivax *(28% vs. 23%) (p = 0.02) and VK 247 (8.1% vs. 4.7%) (p = 0.005) among females, this was not the case for any of the other synthetic peptides.

### Multiplex PCR

**Figure 3:**
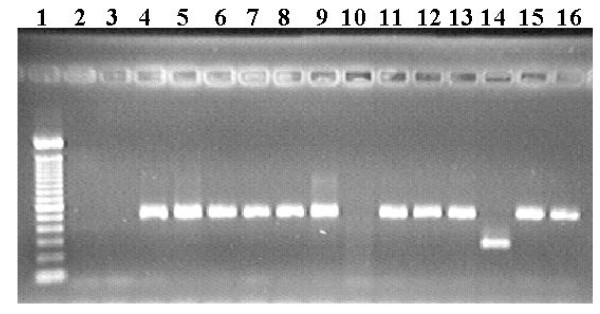
DNA extracted from acute malaria patients and amplified by multiplex PCR. 1: Molecular weight (100 bp, Invitrogen); 2: negative control; 3 and 10: negative results from positive patients; 4–9, 11–13 and 15–16: *P. vivax *fragment (499 bp); 14: *P. malariae *fragment (269 bp).

Multiplex PCR was used for samples from 48 patients. *P. vivax *fragments corresponding to 499 bp were amplified in 45 patients' samples (93.8%), providing evidence of *P. vivax *infection, while a 269-bp fragment corresponding to *P. malariae *infection was detected in one patient's sample (2.1%). No amplification was seen for two patients (4.2%). Some of these results are shown in Figure 3.

**Figure 4:**
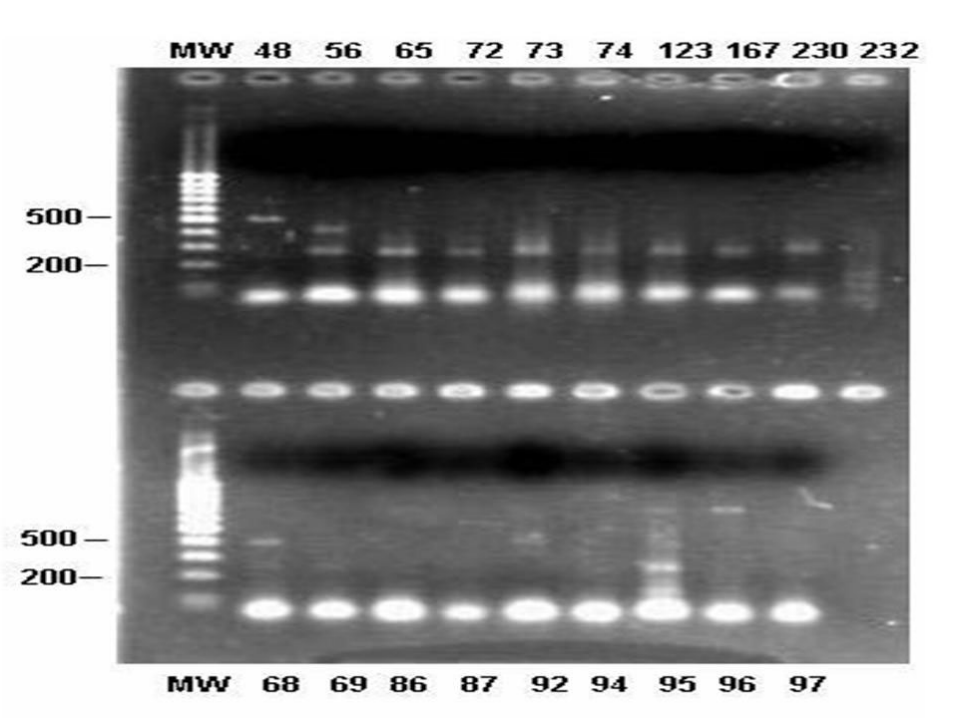
DNA extracted from blood samples collected from the residents of the endemic area in the state of Espírito Santo and amplified by multiplex PCR. Numbers in the columns represent numbers of the samples. MW: (100 bp, Invitrogen). Upside: 48 = *P. vivax*; 56 = *P. malariae *and *P. falciparum*; 65 to 230 = *P. malariae*. Downside: 68 = *P. vivax*; 95 = *P. malariae*; 69, 86, 87, 92, 94, 96 and 97 = negative samples.

This technique was also used for samples from 1,527 residents (85.9% of the total). *P. vivax *was amplified in 23 samples (1.5%), *P. malariae *in 15 (0.9%) and a 395 bp fragment compatible with *P. falciparum *in 9 (0.5%). Mixed infection (*P. malariae*/*P. falciparum*) was detected in one sample (Figure 4). All residents who were positive by multiplex-PCR were asymptomatic at the time of sampling, and no parasites were identified by either thick or thin blood smears. While this study was being carried out, these subjects did not develop signs or symptoms.

### IFA for detection of anti-blood stage antibodies in residents of the non-endemic area

One hundred ninety-two residents of the non-endemic area provided blood samples for IFA testing. Median age of these subjects was 16 years and the majority were female (56.3%). Median time for which patients had lived in the location was 13 years. About half of them (104/192) (54.7%) had been born in the same area they were living in, and none had been born in malarial areas. Travel outside the Amazon region in the preceding two years was declared by 138 subjects (71.9%). IFA for anti-blood-stage antibodies was negative for all of them.

### Random mosquito captures

During the course of the study, 785 anopheline specimens were captured at the various locations where malaria was endemic. The species identified are shown in Table 3. One 100-bp fragment compatible with *P. vivax *was amplified in a sample containing seven specimens of *An. (N.) evansae*.

**Table 3:** Anopheline mosquitoes captured at the various malaria transmission locations in the endemic area in Espírito Santo.

**Species**	**Number**	**Percentage**
***An. *(*N.*) *evansae***	233	29.7
***An. *(*N.*) *strodei***	193	24.6
***An. *(*N.*) *lutzi***	156	19.9
***An. *(*N.*) *argyritarsis***	88	11.2
***An. *(*N.*) *albitarsis***	27	3.4
***An. *(*N.*) *galvaoi***	22	2.8
***An. *(*K.*) *cruzii***	16	2
***An. *(*N.*) *minor***	8	1
***An. *(*N.*) *parvus***	4	0.5
***An. *(*N.*) *triannulatus***	4	0.5
***Anopheles sp.***	34	4.3

**Total**	785	100

The percentages do not give a total of 100% because of rounding.

## Discussion

This cross-sectional survey investigated 65 patients out of a total of 70 cases registered in a period of three years in an area of very low malaria incidence. The results revealed an absolute predominance of infections by all the CSP variants of *P. vivax*. There was no case clustering that could suggest transmission between patients, except possibly patients 42, 44 and 45. As the cases occurred very far from each other, the possibility of a simple man-vector-man transmission cycle must be questioned. Most of the patients were males, and scattered mosquito captures in the area surrounding their houses identified anopheline species with a limited potential for transmission such as *An. evansae*, *An. lutzi *and *An. strodei*, all of which are considered to have limited anthropophilic behaviour [16-18]. Taken together, these data strongly suggest outdoor transmission as most of the work on the land, where the probability of exposure is greater, is performed by males, and there are no vectors which could cause transmission inside the houses.

Two possible explanations for transmission in such a setting may be considered. The first one is that the cases detected are only the tip of a greater number of human infections that are either manifested as self-limited diseases or remain asymptomatic. In fact, a population-based survey conducted simultaneously in the area disclosed a significant prevalence of antibodies against the blood-stage forms and a small number of asymptomatic persons with positive PCR results. A significant positive correlation was found between the length of time for which the subjects had been living in the endemic area and IgG antibodies against *P. vivax *in the IFA test but not between the same demographic variable and other IFA test results. The same results for *P. vivax *antibodies were described by Curado et al. [9] in other areas covered by the Atlantic Forest. The discrepancy observed for other IFA tests could be explained by the smaller sample size of the subjects tested for anti-*P. malariae *and anti-*P. falciparum *antibodies. Nevertheless, it is not clear why there are so few symptomatic persons if about forty percent of the resident subjects have evidence of previous infection according to the results of IFA. While one can argue that the IFA test is not very specific, previous studies [10,19] revealed an acceptable specificity for the IFA test, which has been used in several serological surveys in endemic areas [20-23]. Furthermore, none of the samples of residents of the non-endemic area in this study gave false-positive results. It is unlikely that symptomatic persons could remain undetected as there is a rigid surveillance of malaria cases in the affected area, and government-provided services are the only source of specific treatment. While it is possible that symptomatic persons could go undetected if their disease was self-limited, this is unlikely in an area of low incidence in view of the absence of a previous history of malaria in the majority of the patients. As the anophelines captured at random in the vicinity of the houses have limited anthropophilic behaviour, the question also arises as to where the vectors are that are so competent at maintaining such a broad asymptomatic reservoir with so few circulating parasites.

The second possible explanation is that there is a non-human parasite reservoir in this area, simian malaria being the strongest possibility. Simian malaria in Brazil is caused by two Plasmodia species, namely *P. brasilianum *and *P. simium *[24-31]. *P. brasilianum *is found both inside the Amazon region and in the South and Southeast of the country [25-32] whereas *P. simium *has a more restricted distribution limited by the 20° South and 30° South latitudes and is only found in the South and Southeast of the country [25,30]. The vector responsible for transmission of both species outside the Amazon region is *Anopheles (K.) cruzii*, the same vector that was responsible for human malaria transmission before the campaigns that eradicated malaria outside the Amazon basin [24-31].

*Plasmodium brasilianum *is considered the same species as human *P. malariae *because of molecular similarities [33,34]. Similarly, *P. simium *has been identified as the Old-World subtype of *P. vivax *circulating in New World monkeys [35]. The same parasites, transmitted by the same vector and circulating in two different host species in the same region, make interspecies transmission possible, at least occasionally. The surveys carried out by Fandeur et al. [34] in humans and simians in French Guyana strongly suggest such a possibility, which was clearly demonstrated in another context with another species, namely *P. knowlesi *[36]. Based on these reports, blood from five patients was assayed by PCR techniques advocated by Li et al. [35]. The preliminary findings regarding this autochthonous malaria showed that *P. vivax *variants corresponding to Old and New World species exist sympatrically in these areas.

One possible explanation is that males in the state of Espírito Santo are occasionally bitten by mosquitoes carrying sporozoites from parasites originally present in simian hosts. The evidence for this fortuitous exposure can readily be seen when one considers that no relationship could be established between patients' serological titres and environmental exposure, suggesting that the infection occurs virtually by chance. Although P. *vivax *IgM titres measured by IFA were greater in young individuals, IgM-positive subjects were found in all age groups (only one patient was IgM negative), suggesting, once more, the fortuitous nature of this infectious process as first infections occurred at different ages. In this context, the low parasite counts could be considered the result of human infections with parasites that are not adapted to humans. The high percentage of seropositive individuals in the population, as well as the small number of PCR-positive asymptomatic subjects, could represent infections that occur in less-exposed people submitted to fewer infective bites and, as a consequence, smaller inocula. Small inocula produced by non-adapted parasites would result in the failure of these same parasites to multiply sufficiently to cause disease, resulting in asymptomatic infections.

One can argue that the opportunity of transmission is lacking. In fact, most of the patients denied contact with the forest. However, when we submitted the location of every case to the Global Positioning System (GPS) and crossed the data with the distribution of the forested areas, there was a perfect match, supporting the assumption that either there was information bias regarding the contact with the forests or the forests were sufficiently close to the agricultural areas to allow interspecies transmission.

During the present study it was possible to screen the blood of four monkeys kept at a centre for the reintroduction of captive animals to the wild and the blood of a wild monkey temporarily maintained at a state facility as a result of an accidental injury (all of the monkeys were *Alouatta guariba *specimens). Simian malaria has already been described in this area [29,37], and one of the four captive animals and the wild specimen were positive both in the blood-smear tests and in the multiplex PCR for *P. malariae/brasilianum*, confirming that simian malaria is still present in the region.

In addition, the samples of two patients tested negative in multiplex PCR. Fandeur et al. [34], in their study in French Guiana, also had a similar finding of no detectable PCR product in samples from positive monkeys with low levels of parasitaemia. Since the reaction was able to amplify other blood samples with similarly low parasite counts, they hypothesized that the failure to detect parasites by PCR in animals that were positive in microscopy might be due to sequence variants in the genes studied in regions crucial for primer binding. They questioned the possibility of the existence of still unrecognized parasite species infecting those simians. The same questioning can be applied in the present situation. Since half the patients were negative for antibodies against all synthetic peptides in anti-CSP ELISA, the presence of unrecognized CSP variants is at least possible.

The possibility of malaria introduced by people coming from the Amazon region is remote. ELISA for anti-CSP antibodies was positive for *P. vivax *VK247 in 6.3% of the residents, but 40% were also positive for *P. malariae*. Although Curado et al. [9] described no cross-reaction in their study; it was believed that the possibility of cross-reactivity between these two antigens makes it difficult to establish if all of the 6.3% represent true infection by variant VK 247 or if part of the 6.3% represent false-positive reactions because of infection by *P. malariae *[33]. It is worth noting that 51 samples were only positive for *P. vivax-*like.

Two findings are difficult to interpret. The first is that it is not clear why there were more positive results for females than for males among the samples collected from the residents and tested for anti-CSP antibodies by ELISA. This predominance could have been influenced by some unrecognized factor that affected these findings.

Second, there is the puzzling finding of *P. falciparum *DNA by multiplex PCR in asymptomatic individuals. As *P. falciparum *is a virulent parasite, any infection would be expected to result in full-blown disease in non-immune individuals. The fact that these individuals did not present classical symptomatology may be due to the antigenic variability identified in various *P. falciparum *isolates [38,39]. It has also been observed that in areas of high transmission such as Gabon, the prevalence of asymptomatic *P. falciparum *carriers increases with age [40]. Chronic infections with the same parasite in a low-transmission area of Sudan were reported by Hamad et al. [41]. In riverside populations from Portuchuelo in the state of Rondônia in the Brazilian Amazon, the native inhabitants and oldest inhabitants are those that are less likely to contract malaria [42,43]. Also, the low percentage of subjects who tested positive for this parasite in IFA and the low antibody titres found suggest that transmission is infrequent. In fact, the IFA GMT could represent cross-reactivity to antigens of *P. vivax *and/or *P. malariae*. As Deane [25] and Fandeur et al. [34] have questioned the possibility of the existence of still-unrecognized simian parasite species and as parasites circulating in simian hosts can be very similar to *P. falciparum *[44], it is also possible that those asymptomatic individuals could represent occasional infections by parasites of simian origin. The possibility of false positive results is remote as no other samples infected by *P. falciparum *were processed by PCR in the laboratory at the time of the study, thus ruling out the possibility of any cross-contamination.

All these intriguing possibilities deserve further investigation. This is the first report of a more comprehensive study that includes an ongoing systematic survey of vector distribution and infection, including captures in two strata of the forest (ground and canopy), and a future search for Plasmodia parasites in the wild monkeys of the area. The authors believe that these research activities will be able to clarify if malaria is a true zoonosis in the state of Espírito Santo.

## Conclusion

Malaria in the state of Espírito Santo, Brazil, is caused predominantly by *P. vivax *with all its variants. The low incidence and isolation among patients make a simple man-to-man transmission cycle unlikely. Furthermore, a greater asymptomatic human reservoir cannot be ruled out, and the evidence of outdoor transmission and the simultaneous occurrence of simian infections with the same parasites raise considerations about the possibility of a simian reservoir.

## Authors' contributions

All the authors contributed equally to this work. The authors declare no conflicts of interest.

## References

[B1] U.S. Department of Health and Human Services Centers for Disease Control and Prevention, 2004. A-Z Index: Malaria. About Malaria, Malaria Facts: Global Distribution. http://www.cdc.gov/malaria/facts.htm#WorldMalaria.

[B2] Diretoria Técnica da Gestão, Secretaria de Vigilância em Saúde, Ministério da Saúde (2005).

[B3] World Health Organization (1991). Basic malaria microscopy.

[B4] Lal AA, De la Cruz VF, Campbell GH, Procell PM, Collins WE, McCutchan TF (1988). Structure of the circumsporozoite gene of *Plasmodium malariae*. Mol Biochem Parasitol.

[B5] Arnot DE, Barnwel JW, Tam JP, Nussenzweig V, Nussenzweig RS, Enea V (1985). Circumsporozoite protein of *Plasmodium vivax*: gene cloning and characterization of the immunodominant epitope. Science.

[B6] McCutchan TF, Lal AA, Cruz VF, Miller LH, Maloy WL, Charoenvit Y, Beaudoin RL, Guerry P, Wistar R, Hoffman SL, Hockmeyer WT, Collins WE, Wirth D (1985). Sequence of the immunodominant epitope for the surface protein on sporozoites of *Plasmodium vivax*. Science.

[B7] Qari SH, Shi Y, Goldman IF, Udhayakumar V, Alpers MP, Collins WE, Lal A (1993). A: Identification of *Plasmodium vivax*-like human malaria parasite. Lancet.

[B8] Zavala F, Tam JP, Masuda A (1986). Synthetic peptides as antigens for the detection of humoral immunity to *Plasmodium falciparum *sporozoites. J Immunol Methods.

[B9] Curado I, Duarte AMRC, Lal AA, Oliveira SG, Kloetzel JK (1997). Antibodies anti-bloodstream and circumsporozoite antigens (*Plasmodium vivax *and *Plasmodium malariae/P. brasilianum*) in areas of very low malaria endemicity in Brazil. Mem Inst Oswaldo Cruz.

[B10] Ferreira AW, Sanchez MC (1988). Malária humana: padronização e optimização de testes sorológicos para diagnóstico individual e inquéritos soroepidemiológicos. Rev Inst Med Trop São Paulo.

[B11] Rubio JM, Benito A, Roche J, Berzosa PJ, García ML, Micó M, Edú M, Alvar J (1999). Semi-nested, multiplex polymerase chain reaction for detection of human malaria parasites and evidence of *Plasmodium vivax *infection in Equatorial Guinea. Am J Trop Med Hyg.

[B12] Faran ME, Linthicum KJ (1981). A handbook of the Amazonian species of *Anopheles*(*Nyssorhynchus*) (Diptera: Culicidae). Mosq Syst.

[B13] Kimura M, Kaneko O, Liu Q, Zhou M, Kawamoto F, Wataya Y, Otani S, Yamaguchi Y, Tanabe K (1997). Identification of the four species of human malaria parasites by nested PCR that targets variant sequences in the small subunit rRNA gene. Parasit Int.

[B14] Oskam L, Schoone GJ, Kroon CM, Lujan R, Davies JB (1996). Polymerase chain reaction for detecting *Onchocerca volvulus *in pools of blackflies. Trop Med Int Health.

[B15] Win TT, Lin K, Mizuno S, Zhou M, Liu Q, Ferreira MU, Tantular IS, Kojima S, Ishii A, Kawamoto F (2002). Wide distribution of *Plasmodium ovale *in Myanmar. Trop Med Int Health.

[B16] Faran ME (1980). Mosquito studies (Diptera, Culicidae). XXXIV. A revision of the Albimanus Section of the subgenus Nyssorhynchus of Anopheles. Contrib Am Entomol Inst (Ann Arbor).

[B17] Guimarães AE, Gentile C, Lopes CM, Sant'Anna A, Jovita AM (2000). Ecology of mosquitoes (Diptera: Culicidae) in areas of Serra da Bocaina National Park, Brazil. I – Habitat distribution. Rev Saude Publica.

[B18] Marrelli MT, Floeter-Winter LM, Malafronte RS, Tadei WP, Lourenço-de-Oliveira R, Flores-Mendoza C, Marinotti O (2005). Amazonian malaria vectors anopheline relationships interpreted from ITS2 rDNA sequences. Med Vet Entomol.

[B19] Abramo C, Fontes CJF, Krettli AU (1995). Cross-reactivity between antibodies in the sera of individuals with leishmaniasis, toxoplasmosis, and Chagas' disease and antigens of the blood-stage forms of *Plasmodium falciparum *determined by indirect immunofluorescence. Am J Trop Med Hyg.

[B20] Biswas S, Saxena QB, Roy A (1990). The natural occurrence of circulating antibodies in populations of endemic malarious areas. Indian J Malariol.

[B21] Jeffery GM, Mcwilson W, Collins WE, Lobel H (1975). Application of the indirect fluorescent antibody method in a study of malaria endemicity in Mato Grosso, Brazil. Am J Trop Med Hyg.

[B22] Carvalho ME, Glasser CM, Ciaravolo RMC, Etzel A, Santos LA, Ferreira CS (1988). Sorologia de malária vivax no foco de Aldeia dos Índios, município de Peruíbe, estado de São Paulo, 1984 a 1986. Cad Saúde Pub.

[B23] de Arruda M, Nardin EH, Nussenzweig RS, Cochrane AH (1989). Sero-epidemiological studies of malaria in indian tribes and monkeys of the Amazon basin of Brazil. Am J Trop Med Hyg.

[B24] Deane LM (1969). Plasmodia of monkey and malaria eradication in Brazil. Rev Lat-Amer Microbiol Parasit.

[B25] Deane LM (1992). Simian malaria in Brazil. Mem Inst Oswaldo Cruz.

[B26] Deane LM, Neto JAF (1969). Malaria em macacos do estado do Rio Grande do Sul. Observações preliminares. Rev Inst Med Trop São Paulo.

[B27] Deane LM, Deane MP, Neto JF (1966). Studies on transmission of simian malaria and on a natural infection of man with *Plasmodium simium *in Brazil. Bull World.

[B28] Deane LM, Deane MP, Neto JF (1967). Estudios sobre la transmision de la malaria simica y sobre una infeccion natural del hombre por *Plasmodium simium *en el Brasil. Bol Sanit Panam.

[B29] Deane LM, Neto JF, Sitônio JG (1968). Novo hospedeiro natural do *Plasmodium simium *e do *Plasmodium brasilianum*: o mono, *Brachyteles arachnoides*. Rev Inst Med Trop São Paulo.

[B30] Deane LM, Neto JAF, Okumura M, Ferreira MO (1969). Malaria parasites of Brazilian monkeys. Rev Inst Med Trop São Paulo.

[B31] Deane LM, Deane MP, Neto JAF, Almeida FB (1971). On the transmission of simian malaria in Brazil. Rev Inst Med Trop São Paulo.

[B32] Arruda ME (1985). Presença do *Plasmodium brasilianum *em macacos capturados na área de enchimento do reservatório da usina hidrelétrica de Tucuruí, Pará. Mem Inst Oswaldo Cruz.

[B33] Cochrane AH, Nardin EH, De Arruda M, Maracic M, Clavijo P, Collins WE, Nussenzweig RS (1990). Widespread reactivity of human sera with a variant repeat of the circumsporozoite protein of *Plasmodium vivax*. Am J Trop Med Hyg.

[B34] Fandeur T, Volney B, Peneau C, De Thoisy B (2000). Monkeys of the rainforest in French Guiana are natural reservoirs for *P. brasilianum*/*P. malariae *malaria. Parasitology.

[B35] Li J, Collins WE, Wirtz RA, Rathore D, Lal A, Mccutchan TF (2001). Geographic subdivision of the range of the malaria parasite *Plasmodium vivax*. Emerg Infect Dis.

[B36] Singh B, Sung LK, Matusop A, Radhakrishnan A, Shamsul SSG, Cox-Singh (2004). A large focus of naturally acquired *Plasmodium knowlesi *infections in human beings. Lancet.

[B37] Deane LM, Neto JAF, Sitônio JG (1968). Estudos sobre malária no estado do Espírito Santo. Rev Bras Biol.

[B38] Rich SM, Ferreira UM, Ayala FJ (2000). The origin of antigenic diversity in *Plasmodium falciparum*. Parasitol Today.

[B39] Awadalla P, Walliker D, Babiker H, Mackinnon M (2001). The question of *Plasmodium falciparum *population structure. TropenMed Parasit.

[B40] Kun JFJ, Missinou MA, Lell B, Sovric M, Knopp H, Bojowald B, Dangelmaier O, Kremsner PG (2002). New emerging *Plasmodium falciparum *genotypes in children during the transition phase from asymptomatic parasitemia to malaria. Am J Trop Med Hyg.

[B41] Hamad AA, El Hassan IM, El Khalifa AA, Ahmed GI, Abdelrahim SA, Theander TG, Arnot DE (2000). Chronic *Plasmodium falciparum *infections in an area of low intensity malaria transmission in the Sudan. Parasitol.

[B42] Camargo EP, Alves F, Silva LHP (1999). Symptomless *Plasmodium vivax *infections in native Amazonians. Lancet.

[B43] Camargo LMA, Noronha E, Salcedo JMV, Dutra AP, Krieger H, Pereira da Silva LH, Camargo EP (1999). The epidemiology of malaria in Rondônia (Western Amazon region, Brazil): study of riverine population. Acta Trop.

[B44] Escalante AA, Ayala FJ (1994). Phylogeny of the malarial genus Plasmodium derived from rRNA gene sequences. Proc Natl Acad Sci USA.

